# Soil bacterial communities of three types of plants from ecological restoration areas and plant-growth promotional benefits of *Microbacterium invictum* (strain X-18)

**DOI:** 10.3389/fmicb.2022.926037

**Published:** 2022-08-05

**Authors:** Chao Liu, Jiayao Zhuang, Jie Wang, Guohua Fan, Ming Feng, Shutong Zhang

**Affiliations:** ^1^Collaborative Innovation Center of Sustainable Forestry in Southern China of Jiangsu Province, Nanjing Forestry University, Nanjing, China; ^2^China National Chemical Construction Investment Group Co., Ltd, Beijing, China

**Keywords:** phytoremediation, bacterial communities, soil nutrient, sequencing technology, bioinoculant, plant-growth promotion

## Abstract

Microbial-assisted phytoremediation promotes the ecological restoration of high and steep rocky slopes. To determine the structure and function of microbial communities in the soil in response to changes in soil nutrient content, the bacterial communities of rhizospheric soil from three types of plants, i.e., *Robinia pseudoacacia*, *Pinus massoniana*, *and Cynodon dactylon*, were analyzed using Illumina sequencing technology. High-quality sequences were clustered at the 97% similarity level. The dominant genera were found to be *RB41*, *Gemmatimonas*, *Sphingomonas*, *Bradyrhizobium,* and *Ellin6067*. The Tukey HSD (honestly significant difference) test results showed that the abundance of *RB41* and *Gemmatimonas* were significantly different among three types of plants (*p < 0.01*). The relative abundances of *RB41* (13.32%) and *Gemmatimonas* (3.36%) in rhizospheric soil samples from *R. pseudoacacia* were significantly higher than that from *P. massoniana* (0.16 and 0.35%) and *C. dactylon* (0.40 and 0.82%), respectively. The soil chemical properties analyses suggested that significant differences in rhizospheric soil nutrient content among the three plant types. Especially the available phosphorus, the content of it in the rhizospheric soil of *R. pseudoacacia* was about 280% (*P. massoniana*) and 58% (*C. dactylon*) higher than that of the other two plants, respectively. The soil bacterial communities were further studied using the correlation analysis and the Tax4Fun analysis. A significant and positive correlation was observed between *Gemmatimonas* and soil nutrient components. Except total nitrogen, the positive correlation between *Gemmatimonas* and other soil nutrient components was above 0.9. The outcomes of these analyses suggested that *Gemmatimonas* could be the indicator genus in response to changes in the soil nutrient content. Besides, the genes involved in metabolism were the major contributor to soil nutrients. This study showed that soil nutrients affect the soil bacterial community structure and function. In addition, pot experiments showed that *Microbacterium invictum* X-18 isolated from the rhizospheric soil of *R. pseudoacacia* significantly improved soil nutrient content and increased *R. pseudoacacia* growth. A significant increase in the numbers of nodules of *R. pseudoacacia* and an increase of 28% in plant height, accompanied by an increase of 94% in available phosphorus was measured in the *M. invictum* X-18 treatment than the control treatment.

## Highlights

- Studying microbial-assisted phytoremediation by exploring soil bacterial community.
- Response of bacterial community to changes in soil nutrients.
- Combination of sequencing and isolation culture technology.
- *Gemmatimonas* and *Microbacterium invictum* X-18 belong to the similar key indicator taxa.
- *Gemmatimonas* and *Microbacterium invictum* X-18 are beneficial for soil and plants.

## Introduction

Human activities have led to the destruction of the mountains resulting in multiple high and steep rocky slopes, adversely affecting the ecological environment and landscape (Wang et al., 2020; Han et al., 2021). Thus, the restoration of the ecological environment in these areas is gaining considerable momentum. However, fragile ecological functions and poor soil nutrient conditions in these areas hinder the long-term and stable survival of plants. Thus, phytoremediation in these areas is challenging. External-soil spray seeding is widely used in such areas for ecological restoration, even then, the fertility of the soil substrate is limited, and failures of ecological restoration are not uncommon. In order to improve soil conditions on high and steep rocky slopes, microbial-assisted phytoremediation is being explored.

Soil microbes are active components of processes such as litter decomposition and soil mineralization. Certain microbes can improve soil quality, promote plant growth, and enhance phytoremediation efficiency by accelerating the weathering of rocks and promoting the production of mineral nutrients and phytohormones (Li et al., 2020; Jia et al., 2021; Tian et al., 2021). In addition, previous studies have demonstrated that bacterial community structure and diversity reflect changes in soil ecology (Su et al., 2018). Thus, understanding the changes in the bacterial community structure and the relationship between bacterial community and soil environment is of utmost importance to study microbial-assisted phytoremediation.

Legumes are commonly used as the protective species to restore high and steep rocky slopes. Legumes are highly resistant to stress and can rebuild degraded ecological functions and restore soil nutrient conditions (Sun et al., 2020). To explore the soil microbial community and its possible ecological functions, soil bacterial communities in *Robinia pseudoacacia, Pinus massoniana,* and *C. dactylon* rhizospheric soil from high and steep rocky slopes in the Yueyang, Hunan, China, were analyzed using Illumina sequencing technology that cultivation-independent. In addition, the soil bacteria were screened, and their soil improvement and plant-growth potential were explored.

## Materials and methods

### Sample collection and soil nutrient analysis

The study site is located on the high and steep rocky slopes on the side of Yueyang Avenue in Yueyang, Hunan, China, where external-soil spray seeding was applied to restore the ecological environment 7 years ago (Supplementary Figure S1). The dominant plant species in the experimental site included *R. pseudoacacia* (R), *P. massoniana* (P), and *C. dactylon* (C), all of which are commonly used plants in phytoremediation. The rhizospheric soil was collected, and three samples (each sample at least 5–10 m away from other plants) were collected for each plant type. Each sample was transported to the laboratory for processing within 24 h. Semi-micro Kjeldahl method was used to determine total nitrogen (TN; Liu et al., 1996; Falco and Magni, 2004), molybdenum-antimony anti-colorimetric method with NaOH melting was used to determine total phosphorus (TP), flame photometry with NaOH melting was used to determine total potassium (TK), alkaline hydrolysis diffusion method was used to determine hydrolyzed nitrogen (HN), molybdenum-antimony anti-colorimetric method with NaHCO_3_ extraction to determine available phosphorus (AP), flame photometry with NH_4_OAc extraction to determine available potassium (AK), and pH meter to determine pH.

### DNA extraction and sequencing

Soil samples were sieved through a 0.075 mm screen, ground, and then lysed using the soil homogenate. Microbial DNA was extracted using the HiPure Soil DNA Kits (Magen, Guangzhou, China) according to the manufacturer’s protocols. The 16S rDNA target region of the ribosomal RNA gene was amplified *via* PCR (95°C for 5 min, followed by 30 cycles at 95°C for 1 min, 60°C for 1 min, and 72°C for 1 min, and a final extension at 72°C for 7 min) using 341F primer (5’-CCTACGGGNGGCWGCAG-3′) and 806R primer (5′- GGACTACHVGGGTATCTAAT-3′) targeting the V3-V4 region. PCR reactions were performed in triplicates. 50 μl PCR mixture containing 10 μl of 5 × Q5@ Reaction Buffer, 10 μl of 5 × Q5@ High GC Enhancer, 1.5 μl of 2.5 mm dNTPs, 1.5 μl of each primer (10 μm), 0.2 μl of Q5@ High-Fidelity DNA Polymerase, and 50 ng of template DNA. Related PCR reagents were from New England Biolabs, United States. AMPure XP beads were used to purify the second round of amplified products. ABI StepOnePlus Real-Time PCR System (Life Technologies, produced in the United States) was used for quantification, and sequencing was performed on the computer based on the PE250 mode pooling of Novaseq 6000.

### Processing of sequencing data

Raw reads were assigned to samples based on their unique barcode, and the barcode sequence was truncated using the lima application (version 2.0.1) of the Pbbioconda package (Pacific Biosciences). This was followed by the analysis of subreads to generate CCS (Circular Consensus Sequencing) reads using PacBio’s open-source software suite Smart Link (version 7.0) with the following parameters: minfullpass = 3, minPredictedAccuacy = 0.99. Primer sequences were trimmed using cutadapt (version 2.10; Martin, 2011). The files generated by the PacBio platform were then used for amplicon size filtering to remove sequences outside the expected amplicon size (minlength 1.3 kb, maxlength 1.7 kb). Reads with the same continuous base number > 8 were considered low-quality reads and thus removed. The clean reads thus obtained were clustered into operational taxonomic units (OTUs) of ≥97% similarity using UPARSE (version 9.2.64) pipeline (Edgar et al., 2011). All chimeric reads were removed using the UCHIME algorithm to obtain clean reads for further analysis. The sequence with the highest abundance was selected as a representative sequence within each cluster.

### Soil microbes’ isolation, identification, and growth-promoting benefits

To study the beneficial microbes in the soil, culturable strains were isolated from the rhizospheric soil of *R. pseudoacacia* with relatively high soil nutrients. Soil samples were diluted and smeared on NA (Nutrient Agar). The agar plates were incubated at 28°C for 3 days. Morphologically distinct colonies were further sub-cultured to obtain a pure culture. The pure cultures were maintained on NA slants at 4°C in a refrigerator.

Since the sample site was phosphorus-deficient, bacterial cultures from these samples were screened for phosphorus solubilizing function. All strains were inoculated on *Monkina* agar at 28°C. The agar plates inoculated with phosphate solubilizing strains exhibited the formation of halos (zone of solubilization) around the colonies after 7 days of incubation. The diameter of the halos (D) around the microbial colonies (d) was measured, and D/d was calculated. Based on D/d, five phosphate solubilizing strains were selected for pot experiments.

To explore the growth-promoting benefits of soil microbes, the phosphate solubilizing bacteria were inoculated into the seedlings of *R. pseudoacacia*, respectively. The seeds of *R. pseudoacacia* plants were surface-sterilized and allowed to germinate for 3 days at 20°C and relative humidity of 60%. Later, the three healthy seedlings were planted in each pot, including mixed matrix soil (Jiangsu Xingnong Matrix Technology Co., Ltd). One month later, only one seedling was allowed to grow per pot, and it was ensured that the growth of seedlings in each pot was identical. Bacterial inoculum was prepared by culturing bacteria in NA. Colonies of each strain were added to a 100 ml Erlenmeyer flask containing 30 ml LB (Luria-Bertani) broth (10 g peptone, 5 g yeast, 5 g NaCl, 1,000 ml deionized water, pH 7.2), incubated at 30°C, and 180 r/min for 12 h. The absorbance of the bacterial suspension was measured (UV-8000 T, Shanghai Metash Instruments Co., Ltd) at 600 nm (18). Inoculum cultures were adjusted to get a final absorbance of 0.8 ~ 1.2. The inoculums were sealed and stored in a refrigerator at 4°C for later use. For inoculation, the stored suspension of the inocula was diluted 100X, and 60 ml of the resulting diluted sample was applied to the soil. The pots were divided into one control group and five inoculation groups, three replicates for each group, and placed in a greenhouse. Subsequently, the control group was added to the sterile LB broth, and the other inoculation groups, including five bacteria, were added with the same concentration of bioinoculant for each strain, respectively.

Three months later, the plants were sampled and analyzed. For plants, vernier calipers and tape were used to measure the ground diameter and height of the seedlings. Root scanners were used to measure the leaf area (ten upper, middle, and lower leaves were selected from each pot to measure the leaf area). The plants were dried to measure the above-ground and under-ground biomass. For potting soil, the Mettler toledo pH meter was used to measure soil’s pH value (water: soil ratio was 5: 1). The alkaline hydrolysis diffusion method was used to determine HN. The molybdenum-antimony anti-colorimetric method with NaHCO_3_ extraction was used to determine AP. The strain demonstrating the best plant-growth-promoting and soil improvement benefits was selected and was subjected to 16S rDNA analysis and morphological characteristics for identification.

### Statistical analysis

To illustrate the microbial diversity of rhizospheric soil in different vegetation types, the alpha diversity (including Chao1, Simpson, and Shannon indices) was quantified based on OTU abundance. The abundance statistics of each taxonomy were visualized using Krona (version 2.6; Ondov et al., 2011). The stacked bar plot of the community composition was visualized in the R project ggplot2 package (Wickham and Chang, 2015). The pvclust package of R with the default settings was used for cluster analysis using Ward’s cluster method. Heat maps and networks of correlation coefficients were generated using Omicsmart, a dynamic real-time interactive online platform for data analysis^1^. Analysis of function difference between groups was calculated by Welch’s *t*-test, Wilcoxon rank test, Kruskal-Wallis H test, and Tukey’s HSD test in the R project Vegan package (version 2.5.3; Oksanen et al., 2010).

## Results

### Rhizosphere soil nutrient

In the current study, the rhizospheric soil nutrients, i. e., N (nitrogen), P (phosphorus), and K (potassium; Table 1), were analyzed. The results of soil nutrients analyses showed that the N and K content was in normal level, and that of P was slightly lower in rhizospheric soil samples from three plants as per the ‘Environmental Quality Standard for Soil’ of China (Author, n.d.). It indicated that soil in sample sites was P deficient. In general, the total rhizospheric soil nutrient content from *R. pseudoacacia* was higher than that of *P. massoniana* and *C. dactylon*. The available rhizospheric soil nutrient content of *R. pseudoacacia* was significantly higher than that of *P. massoniana* and *C. dactylon*. Different plant species exhibited different rhizospheric soil nutrient content, and the rhizospheric soil nutrient content of *R. pseudoacacia* was higher than other plant species.

**Table 1:** The rhizospheric soil nutrient content of *Robinia pseudoacacia*, *Pinus massoniana*, *and Cynodon dactylon*.

Samples	TN	TP	TK	HN	AP	AK	pH
	(g/kg, dry weight)			(mg/kg, dry weight)			
*R. pseudoacacia*	3.65 ± 0.04a	0.13 ± 0.01a	26.78 ± 0.54a	241.32 ± 38.23a	0.057 ± 0.006a	162.48 ± 1.21a	5.65 ± 0.22a
*P. massoniana*	1.90 ± 0.08b	0.07 ± 0.01b	21.37 ± 0.69ab	136.32 ± 16.64b	0.015 ± 0.001c	110.87 ± 1.10c	5.08 ± 0.17b
*C. dactylon*	3.13 ± 0.06a	0.10 ± 0.01a	23.13 ± 0.56a	196.84 ± 23.19ab	0.036 ± 0.001b	135.68 ± 1.10b	5.12 ± 0.13ab

The values “a, b, c” represent the standard deviation. Different letters represent significant differences. TN, Total nitrogen; TP, Total phosphorus; TK, Total potassium; HN, Hydrolyzable nitrogen; AP, Available phosphorus; and AK, Available potassium.

### Effects of soil nutrients on bacterial communities

The bacterial communities of nine rhizospheric soil samples were analyzed using Illumina sequencing technology. The atypical rhizospheric soil samples collected from *R. pseudoacacia* were removed. The dataset of eight samples consisted of 837,739 unique 16S rDNA gene tags (Table 2). The OTUs per sample ranged from 2088 to 2,542. The α-diversity (Chao 1, Shannon, and Simpson) analysis assessed the bacterial abundance in rhizospheric soil samples (Table 2). Chao1 index evaluated the total number of OTUs in all the rhizospheric soil samples, demonstrating the species richness of the microbial communities. Shannon and Simpson indices reflected the richness and uniformity of the microbial communities and indicated that the α-diversity of both *R. pseudoacacia* and *C. dactylon* was higher than that of *P. massoniana*, which had a lower soil nutrient content.

**Table 2:** α-diversity of bacteria from the soil samples *distance < 0.03*.

Samples	Unique tags	Number of OTUs	α-diversity		
			Chao1	Shannon	Simpson
*R. pseudoacacia*	118,357	2,447	2,622	8.67	0.9907
*P. massoniana*	90,533	2,233	2,495	8.66	0.991
*C. dactylon*	109,808	2,506	2,722	9.02	0.9944

**Figure 1:**
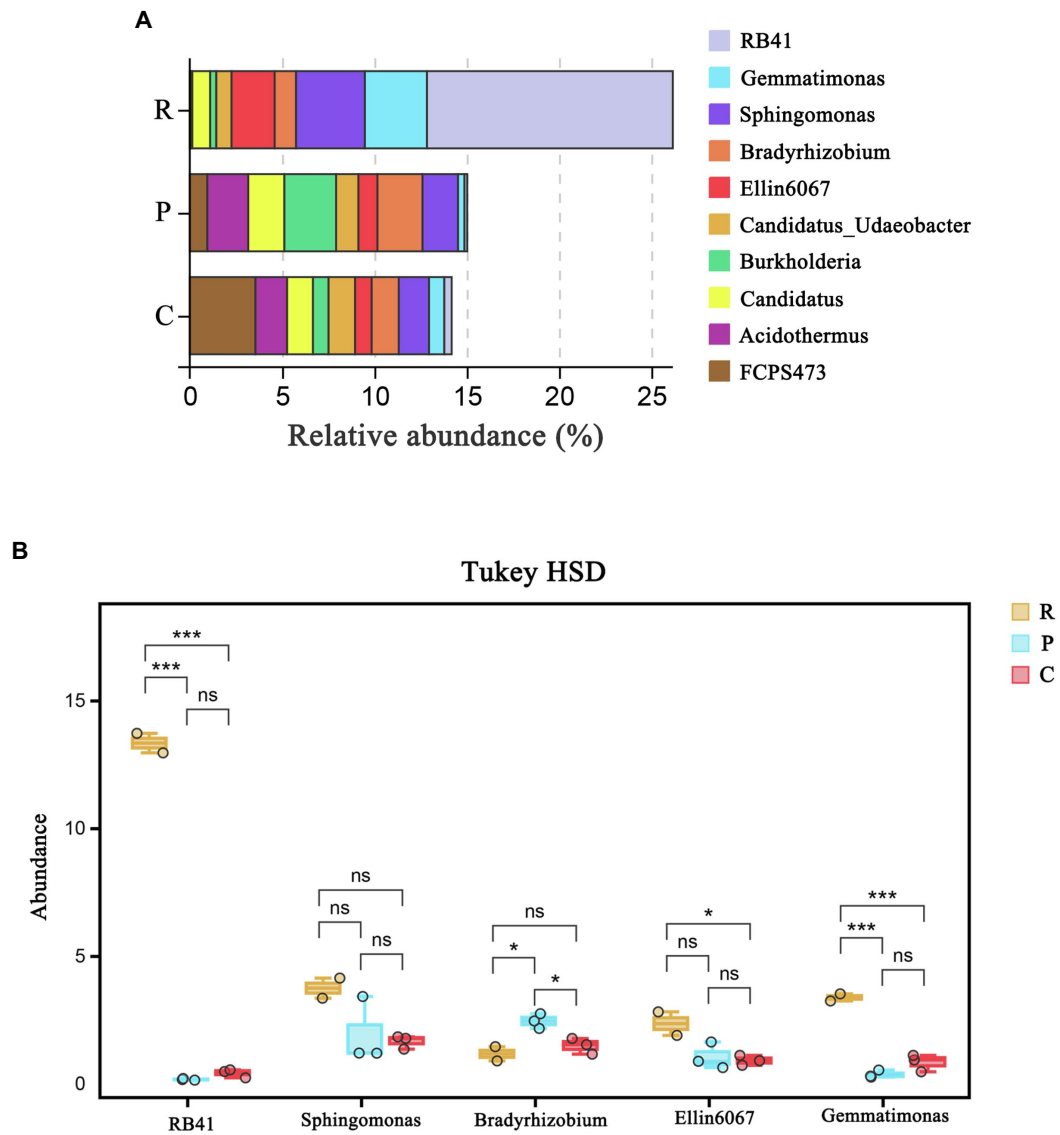
The bacterial community structure at the genus level: **(A)** Tukey HSD test at genus level with 95% confidence intervals and **(B)** ^*^p < 0.05; ^***^p < 0.01. R, P, C represent *Robinia pseudoacacia*, *Pinus massoniana,* and *Cynodon dactylon*, respectively.

Bacterial community structure in soil samples at the genus level is demonstrated in Figure 1A. *RB41*, *Gemmatimonas*, *Sphingomonas*, *Bradyrhizobium,* and *Ellin6067* were found to be the dominant genera in the rhizospheric soil samples. Tukey HSD test method was used to detect the significance of species differences at the genus level (Figure 1B). The relative abundances of *RB41* (13.32%) and *Gemmatimonas* (3.36%) in rhizospheric soil samples from *R. pseudoacacia* were significantly higher than that from *P. massoniana* (0.16 and 0.35%) and *C. dactylon* (0.40 and 0.82%), respectively. Furthermore, the relative abundances of *RB41* and *Gemmatimonas* were significantly different among the three plant types (*p < 0.01*), indicating that *RB41* and *Gemmatimonas* may be correlated to the difference in rhizospheric soil nutrient content.

**Figure 2:**
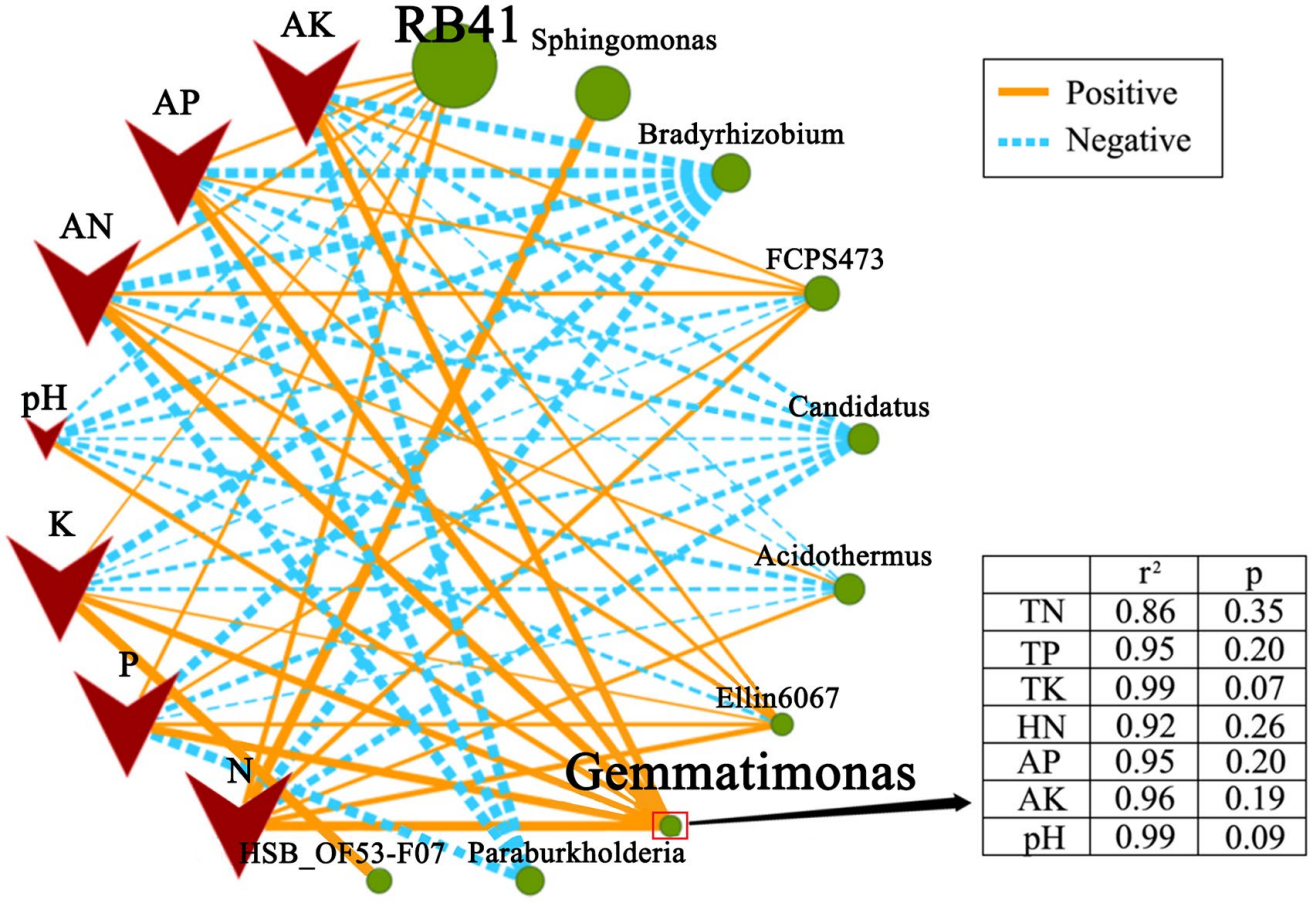
Correlation network map illustrating the correlation between soil nutrient and soil bacterial community at the genus level. Nodes represent genus and soil nutrient, lines represent correlations, and the thickness of the line represents the degree of correlation (*0.05 < p < 0.5*).

The correlation analysis validated the correlation between soil nutrients and soil bacterial community (Figure 2). Bacterial genera, *RB41*, *Gemmatimonas*, and *Ellin6067*, were positively correlated to soil nutrient content (*0.05 < p < 0.5*). Compared with *RB41* and *Ellin6067*, a significant and positive correlation was observed between *Gemmatimonas* and soil nutrient components. It indicated that *Gemmatimonas* might be most relevant to the rhizosphere soil nutrient difference.

**Figure 3:**
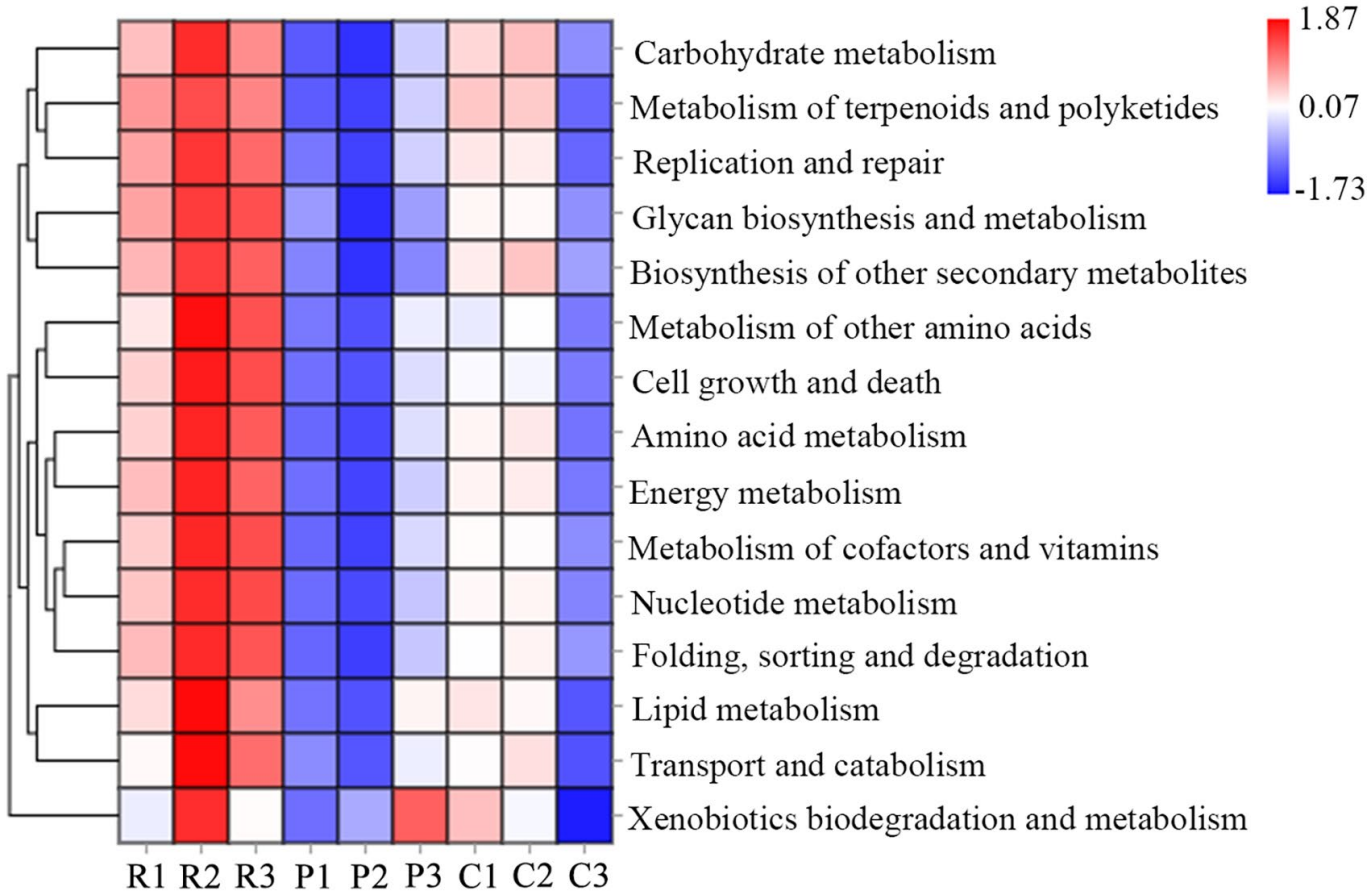
Heat map cluster and abundance of microbial functions at the genus level. R, P, and C represent *Robinia pseudoacacia*, *Pinus massoniana,* and *Cynodon dactylon*, respectively.

The functional abundance of bacterial communities of rhizospheric soil from three types of plants were identified using 16S rDNA gene amplicon data and Tax4Fun (Figure 3). Several predicted pathways were significantly enriched (*p < 0.5*) in the bacterial communities of rhizospheric soil from *R. pseudoacacia*, specifically genes associated with metabolism (carbohydrate metabolism, amino acid metabolism, energy metabolism, biosynthesis of another secondary metabolism, and so on), genetic information processing (replication and repair, folding, sorting, and degradation), and environmental information processing (cell growth and death, transport, and catabolism). In addition, almost all the secondary sub-classification pathways in metabolism were significantly enriched in the *R. pseudoacacia* rhizospheric soil bacterial communities. Besides, the abundance of bacterial metabolic functions in the rhizospheric soil of different plants was distinct due to differences in nutrient content. The results showed that vigorous microbial metabolism is associated with a higher soil nutrient content.

The 16S sequence of rhizospheric soil samples was deposited in the NCBI (The National Center for Biotechnology Information) database with the number PRJNA757370.

### Isolation, identification, and benefits of X-18 of soil microbes

Since the study site was phosphorus-deficient, phosphate solubilizing bacteria were screened in the current study. As shown in Table 3, five phosphate solubilizing bacteria were screened from the rhizospheric soil of *R. pseudoacacia*.

**Table 3:** Phosphorus-dissolving effect of phosphate solubilizing bacteria.

Name	D/d (organic phosphorus)	D/d (inorganic phosphorus)
X-4	3.61	2.13
X-8	/	2.60
X-11	3.06	4.54
X-14	3.47	2.81
X-18	3.62	2.87

**Figure 4:**
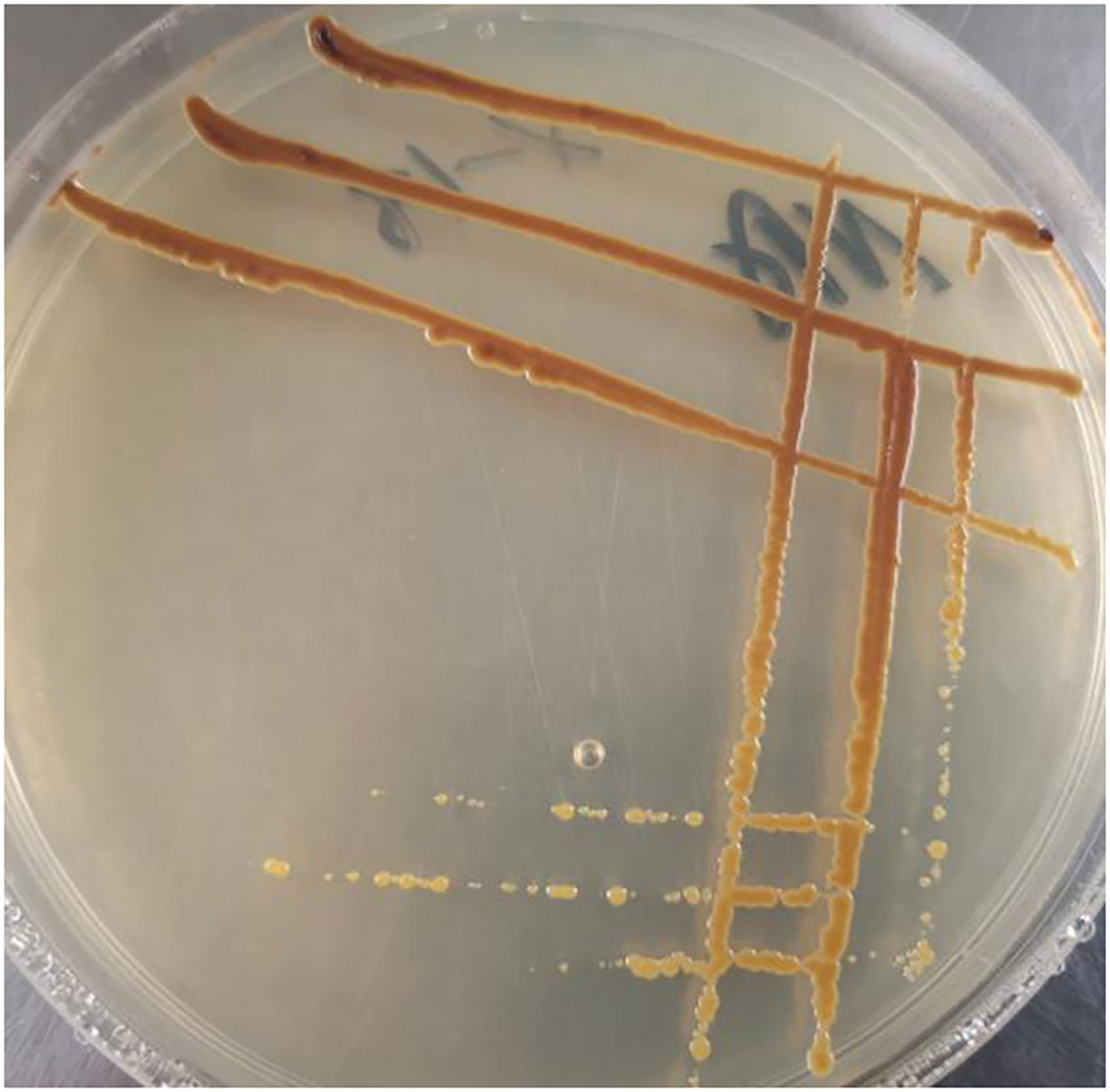
*Microbacterium invictum* X-18.

According to the results of the pot experiment, X-18 exhibited the highest efficacy for plant-growth promotion. X-18 was identified as *Microbacterium invictum* (Figure 4; accession number MN586282). The X-18 isolate was deposited at the CCTCC (China Center for Type Culture Collection) with the accession number M2019236. Plant growth characteristics and soil nutrient concentration are demonstrated in Table 4. Compared with the control, the dry weight of the root of the X-18 inoculated plants significantly increased by 122%, ground diameter by 22%, plant height by 28%, and average leaf area by 18%. In addition, the number and total weight of nodules also increased significantly. With a significant increase of 55.34 and 93.67% (*p < 0.05*) compared to the control, HN and AP of potted soil inoculated with X-18 were 113.52 mg/kg and 2.599 mg/kg, respectively.

**Table 4:** Effects of X-18 on plant and soil.

Groups (Sample)	Soil (potted)			Plant (above ground)			Plant (underground)		
	HN (mg/kg)	AP (mg/kg)	pH	Ground diameter (m)	Plant height (m)	Average leaf area (m^2^)	Number of nodules	Total nodules weight(g)	Dry weight(g)
CK	73.08 ± 2.52b	1.342 ± 0.039b	5.35 ± 0.026b	4.75 × 10^−3^ ± 0.07b	40.04 × 10^−2^ ± 1.56c	4.353 × 10^−4^ ± 0.12b	0	/	0.23 ± 0.05b
X-18	113.52 ± 2.61a	2.599 ± 0.022a	5.17 ± 0.015a	5.81 × 10^−3^ ± 0.20a	51.20 × 10^−2^ ± 0.31a	5.131 × 10^−4^ ± 0.19a	5	0.0083 ± 0.0007a	0.51 ± 0.02a

The values “a, b” represent the standard deviation. Different letters represent significant differences. HN, Hydrolyzable nitrogen and AP, available phosphorus.

## Discussion

### Soil nutrient and soil bacterial community

In the present study, the rhizospheric soil from *R. pseudoacacia* was found to contain an increased level of total and available rhizospheric soil nutrients compared to *P. massoniana* and *C. dactylon* (Table 1). Besides, the α-diversity of soil bacteria from *R. pseudoacacia* and *C. dactylon* was higher than that of *P. massoniana* (*p < 0.05*; Table 2). These results are consistent with the previous findings that the diverse bacterial community is the result of the physiological or evolutionary adaptation of microbes to the environment (Wang et al., 2021).

The above analysis indicated that *Gemmatimonas* might be the key genus in response to changes in the soil nutrient content. *Gemmatimonas* demonstrated strong positive associations with soil nutrient transformations, and thus it emerged as the key taxa beneficial for plant growth (Li et al., 2017). According to the previous studies, *Gemmatimonas* can dissolve insolubilized P and convert it into P for plant growth. *Gemmatimonas* was found to be more abundant in the rhizospheric soil of healthy plants than in diseased soil (She et al., 2016), indicating its disease-suppressing properties. Besides, *Gemmatimonas* can solubilize insoluble elements, induce plant stress resistance or produce antifungal antibiotics (Mahanty et al., 2017; Shang and Liu, 2020; Akinola et al., 2021; Zhu et al., 2021). In the present study, *Gemmatimonas* was the dominant genus in the rhizospheric soil from three plant types, accounting for up to 3.36% of all OTUs from *R. pseudoacacia*. However, only 0.35 and 0.82% of all OTUs in the rhizospheric soil from *P. massoniana* and *C. dactylon* were *Gemmatimonas*. The correlation analysis showed that *Gemmatimonas* is significantly and positively correlated with all soil nutrient factors (Figure 3). Overall, if *Gemmatimonas* is beneficial to improving soil nutrients, more experiments will be necessary to understand the functional mechanism of these soil bacteria.

Microbes participating in the process of soil nutrient transformations are all connected through genes (Castaneda and Barbosa, 2017; Chen et al., 2019; Srour et al., 2020). In the present study, Tax4Fun was applied to infer the differences in the dominant functional traits dominating through 16S rDNA gene data. Genes involved in metabolism, including “carbohydrate metabolism,” “amino acid metabolism,” “energy metabolism,” and so on, were significantly more abundant in soil with high nutrients than in low nutrient conditions (Figure 4). It suggested that the function of the microbial community significantly contributed to responses to nutrient changes in the soil environment.

### The benefits of *Microbacterium invictum* X-18

*Microbacterium invictum* X-18 significantly increased the nutrient content of potted soil. Similarly, the number and weight of nodules, root dry weight, plant height, ground diameter, and average leaf area of X-18 inoculated plants significantly increased compared to the control group (*p < 0.05*). Sang et al. (2014) reported that *Microbacterium* sp. exhibited phosphate solubilizing activity to promote plant growth. Ribeiro et al. (2021) also showed that *Microbacterium* sp. can promote plant growth by increasing the production of IAA, siderophore, ACC deaminase, and decarboxylase. These results indicate that the mechanism of *Microbacterium* sp. to promote plant growth may improve soil nutrient content and increase the production of auxins, enzymes, and so on.

Accordingly, in the current study, it revealed that both *M. invictum* X-18 and *Gemmatimonas* belong to the similar taxa, which are beneficial to increase soil nutrient content and plant growth.

## Conclusion

In this study, the rhizospheric soil bacterial community structure from three types of plants based on cultivation-independent Illumina sequencing technology was analyzed. We observed that *Gemmatimonas* is the indicator genus to assess soil nutrient changes. The results showed that the diverse bacterial community is the result of adaptation to environmental changes. *M. invictum* X-18 in soil improvement and plant-growth promotion was validated through the plate culture experiment and pot experiment. Compared with the control, the nodule, dry weight and plant height inoculated with X-18 increased significantly. The current study demonstrated a novel thought of using two approaches to explore key microbial taxa in response to changes in the soil environment. *M. invictum* X-18 strain identified in this study will be beneficial to promote the development of microbial-assisted phytoremediation technology. These work proved the feasibility and efficiency of microbial-assisted phytoremediation. It provides strain and theoretical guidance for improving the phytoremediation efficiency of barren areas.

## Data availability statement

The data presented in the study are deposited in the NCBI repository, accession number PRJNA757370 (https://dataview.ncbi.nlm.nih.gov/object/PRJNA757370).

## Author contributions

JZ enabled and supervised this research and conceived of the study. CL designed the experiments, conducted data analysis, and wrote the manuscript. CL, JW, GF, MF, and SZ performed the experiments. All authors contributed to the article and approved the submitted version.

## Funding

This work was funded by the National Key R&D Program of China (2017YFC0505500 and 2017YFC0505506).

## Conflict of interest

SZ was employed by China National Chemical Construction Investment Group Co., Ltd.

The remaining authors declare that the research was conducted in the absence of any commercial or financial relationships that could be construed as a potential conflict of interest.

## Publisher’s note

All claims expressed in this article are solely those of the authors and do not necessarily represent those of their affiliated organizations, or those of the publisher, the editors and the reviewers. Any product that may be evaluated in this article, or claim that may be made by its manufacturer, is not guaranteed or endorsed by the publisher.

## Footnotes

^1^ http://www.omicsmart.com

## References

[ref1] Akinola S. Ayangbenro A. Babalola O. (2021). Metagenomic insight into the community structure of maize-Rhizosphere Bacteria as predicted by different environmental factors and their functioning within plant proximity. Microorganisms 9:1419. doi: 10.3390/microorganisms90, PMID: 34209383 PMC8304108

[ref2] Author X. (n.d.). Environmental Quality Standard for Soil’ of China (GB15618-2018).

[ref3] Castaneda L. Barbosa O. (2017). Metagenomic analysis exploring taxonomic and functional diversity of soil microbial communities in Chilean vineyards and surrounding native forests. PeerJ 5:e3098. doi: 10.7717/peerj.3098, PMID: 28382231 PMC5376117

[ref4] Chen J. Shen W. Xu H. Li Y. Luo T. (2019). The composition of nitrogen fixing microorganisms correlates With soil nitrogen content During reforestation: A comparison Between legume and non-legume plantations. Front. Microbiol. 10:508. doi: 10.10.3389/fmicb.2019.00508 30930882 PMC6427063

[ref6] Edgar R. Haas B. Clemente J. Quince C. Knight R. (2011). UCHIME improves sensitivity and speed of chimera detection. Bioinformatics 27, 2194–2200. doi: 10.1093/bioinformatics/btr381, PMID: 21700674 PMC3150044

[ref7] Falco G. Magni P. (2004). Sediment grain size and organic carbon distribution in the cabras lagoon (Sardinia, western mediterranean). Chem. Ecol. 20, 367–377. doi: 10.1080/02757540310001629189

[ref8] Han C. Gao Z. Wu Z. Huang J. Liu Z. Zhang L.. (2021). Restoration of damaged ecosystems in desert steppe open-pit coal mines: effects on soil nematode communities and functions. Land Degrad. Dev. 32, 4402–4416. doi: 10.1002/ldr.4045

[ref9] Jia Z. Meng M. Li C. Zhang B. Zhai L. Liu X.. (2021). Rock-solubilizing microbial inoculums have enormous potential as ecological remediation agents to promote plant growth. Forests 12:357. doi: 10.3390/f12030357

[ref10] Li Y. Adams J. Shi Y. Wang H. He J. Chu H. (2017). Distinct soil microbial communities in habitats of differing soil water balance on the Tibetan plateau. Sci. Rep. 7:407. doi: 10.1038/srep46407, PMID: 28401921 PMC5388882

[ref11] Li C. Jia Z. Peng X. Zhai L. Zhang B. Liu X.. (2020). Functions of mineral-solubilizing microbes and a water retaining agent for the remediation of abandoned mine sites. Sci. Total Environ. 761:143215. doi: 10.1016/j.scitotenv.2020.143215, PMID: 33160670

[ref12] Liu G. Jiang N. Zhang L. Liu Z., (1996). Soil Physical and Chemical Analysis and Description of Soil Profiles. Beijing: China Standard Methods Press.

[ref13] Mahanty T. Bhattacharjee S. Goswami M. Bhattacharyya P. Das B. Ghosh A.. (2017). Biofertilizers: a potential approach for sustainable agriculture development. Environ. Sci. Pollut. Res. Int. 24, 3315–3335. doi: 10.1007/s11356-016-8104-0, PMID: 27888482

[ref14] Martin M. (2011). Cutadapt removes adapter sequences from high-throughput sequencing reads. EMBnet J. 17:200. doi: 10.14806/ej.17.1.200

[ref15] Oksanen J. Blanchet F. Kindt R., (2010). Vegan: community ecology package. R package version. R package, version. 23.

[ref16] Ondov B. Bergman N. Phillippy A. (2011). Interactive metagenomic visualization in a web browser. BMC Bioinform. 12:385. doi: 10.1186/1471-2105-12-385, PMID: 21961884 PMC3190407

[ref17] Ribeiro I. D. A. Bach E. Moreira F. Mvller A. R. Rangel C. P. Passaglia L. (2021). Antifungal potential against Sclerotinia sclerotiorum (Lib.) de Bary and plant growth promoting abilities of Bacillus isolates from canola (*Brassica napus* L.) roots. Microbiol. Res. 248:126754. doi: 10.1016/j.micres, PMID: 33848783

[ref18] Sang H. Mayank A. Se C. (2014). Isolation and characterization of plant growth promoting endophytic diazotrophic bacteria from Korean rice cultivars. Microbiol. Res. 169, 83–98. doi: 10.1016/j.micres.06.003, PMID: 23871145

[ref19] Shang J. Liu B. (2020). Application of a microbial consortium improves the growth of Camellia sinensis and influences the indigenous rhizosphere bacterial communities. J. Appl. Microbiol. 130, 2029–2040. doi: 10.1111/jam.14927, PMID: 33170985

[ref20] She S. Niu J. Zhang C. Xiao Y. Chen W. Dai L.. (2016). Significant relationship between soil bacterial community structure and incidence of bacterial wilt disease under continuous cropping system. Arch. Microbiol. 199, 267–275. doi: 10.1007/s00203-016-1301-x, PMID: 27699437

[ref21] Srour A. Ammar H. Subedi A. Pimente M. Cook R. Bond J.. (2020). Microbial communities associated With long-term tillage and fertility treatments in a corn-soybean cropping system. Front. Microbiol. 11:1363. doi: 10.3389/fmicb.01363, PMID: 32670235 PMC7330075

[ref22] Su X. Li Y. Yang B. Li Q. (2018). Effects of plant diversity on soil microbial community in a subtropical forest. J. Ecol. 37, 2254–2261. doi: 10.13292/j.1000-4890.201808.014

[ref23] Sun Q. Liu Y. Liu H. Dumroese R. K. (2020). Interaction of biochar type and rhizobia inoculation increases the growth and biological nitrogen fixation of *Robinia pseudoacacia* seedlings. Forests 11:711. doi: 10.3390/f11060711

[ref25] Tian D. Su M. Zou X. Zhang L. Tang L. Geng Y.. (2021). Influences of phosphate addition on fungal weathering of carbonate in the red soil from karst region. Sci. Total Environ. 755:142570. doi: 10.1016/j.scitotenv.2020.142570, PMID: 33035850

[ref26] Wang M. Liu Q. Pang X. (2020). Evaluating ecological effects of roadside slope restoration techniques: A global meta-analysis. J. Environ. Manag. 281:111867. doi: 10.1016/j.jenvman 33385908

[ref27] Wang S. Zuo X. Awada T. Medima E. Feng K. Yue P.. (2021). Changes of soil bacterial and fungal community structure along a natural aridity gradient in desert grassland ecosystems. Inner Mongolia. Catena 2021:205. doi: 10.1016/j.catena

[ref28] Wickham H. Chang W., (2015). ggplot2: An implementation of the grammar of graphics. http://CRAN.R-project.org/package=ggplot2. R package version 1.

[ref30] Zhu Y. Shao T. Zhou Y. Zhang X. Rengel Z. (2021). 2021. Periphyton improves soil conditions and offers a suitable environment for rice growth in coastal saline alkali soil. Land Degrad. Dev. 32, 2775–2788. doi: 10.1002/ldr.3944

