# Supplementary material for: Soil bacterial communities of three types of plants from ecological restoration areas and plant-growth promotional benefits of Microbacterium invictum (strain X-18)
Source: Front Microbiol. 2022 Aug 5;13:926037. doi: 10.3389/fmicb.2022.926037 (PMC9389310; doi:10.3389/fmicb.2022.926037)
Supplement: Supplementary file 1 [file Data_Sheet_1.docx]

| 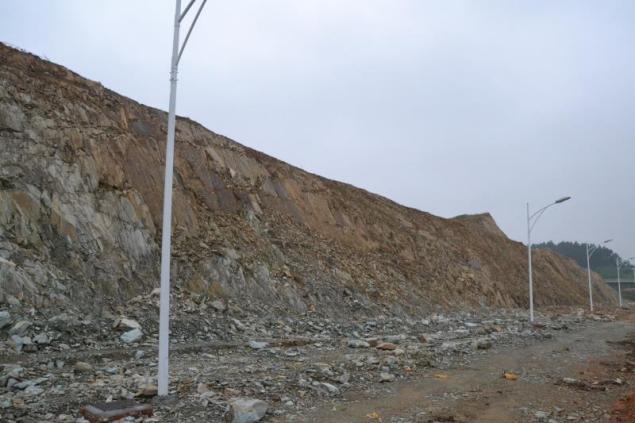 | **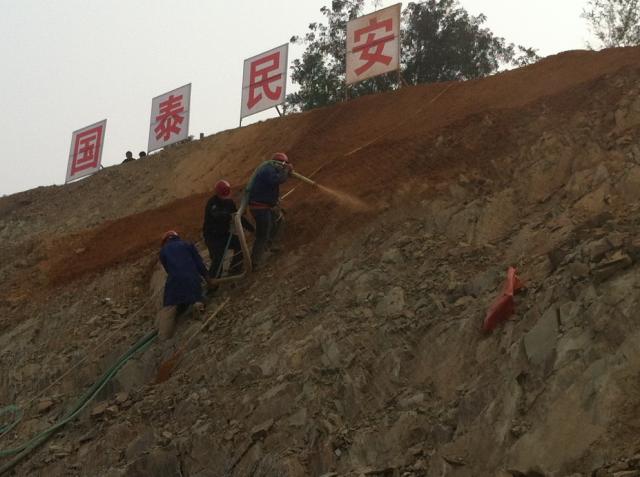** |
| --- | --- |
| **a** | **b** |
| 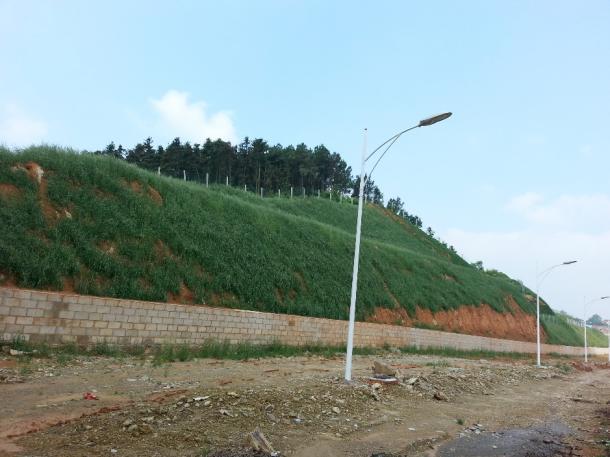 | **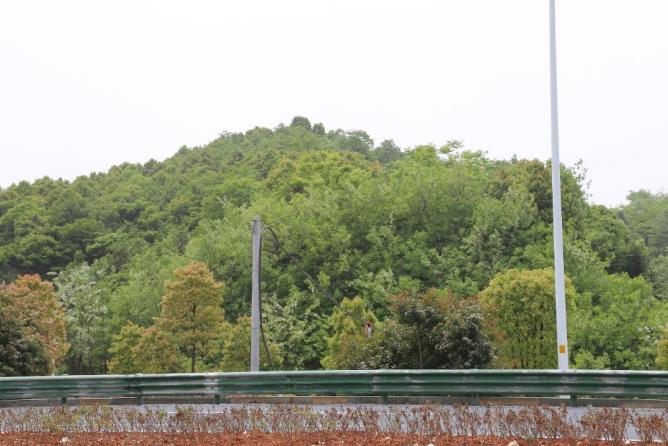** |
| **c** | **d** |
| Fig.S1. Spraying site of high and steep rocky slope on the side of the Yueyang Avenue in Yueyang, Hunan, China. (a) refers to the original appearance before spraying. (b) shown the work about spraying. (c) refers to the appearance of the high and steep rocky slope after spraying for two months. (d) refers to the appearance of the high and steep rocky slope after spraying for five years. | |

The study site is located on the high and steep rocky slopes on the side of Yueyang Avenue in Yueyang, Hunan, China, where external-soil spray seeding was applied to restore the ecological environment seven years ago.
